# Whole blood targeting and activation of monocytes with TLR7 agonist formulated in cationic liposomes

**DOI:** 10.1186/2051-1426-1-S1-P130

**Published:** 2013-11-07

**Authors:** Simon S Jensen, Pia T Johansen, Daniel Zucker, Jonas Henriksen, Thomas Andresen, Alcide Barberis, Roberto Maj, Houman Pourhassan, Jeanette Wern, Monika Gad

**Affiliations:** 1 Immune Cell Targeting, Bioneer, Hoersholm, Denmark; 2 Depertment of Micro and Nanotechnology, DTU Nanotech, Lyngby, Denmark; 3 Telormedix, Bioggio, Switzerland

## 

Monocytes are one of the major phagocytic cells in the periphery that patrols the circulation for invading pathogens, and upon activation differentiates into dendritic cells, capable of migration to lymph nodes eliciting an adaptive immune response. Monocytes has for more than a decade been precursor cell for generation of autologous dendritic cell cancer vaccines, but clinical results have shown limiting benefits for the patients. One way of improving dendritic cell vaccines is targeting the monocytes in vivo with a suitable carrier of adjuvant together with tumor antigens, to boost monocyte differentiation towards tumor antigen presenting DCs. Here we report a novel monocyte targeting liposome technology capable of delivering TLR7 agonist to CD14 positive monocytes in fresh whole human blood. Liposomes with a positive surface charge were able to specifically target monocytes over lymphocytes and granulocytes, and showed association with 90-100 % of the monocytes. Formulation of the TLR7 agonist in monocyte targeting liposomes showed strong activation of the monocytes, with potent induction of proinflammatory cytokines, and differentiation into tissue inflammatory DCs, demonstrating that the liposomes are able to deliver compounds to the endosomes where TLR7 is present. The present monocyte targeting technology may be a promising approach for designing cancer vaccines with suitable adjuvants and cancer antigens.

